# Caudal type homeoboxes as a driving force in *Helicobacter pylori* infection-induced gastric intestinal metaplasia

**DOI:** 10.1080/19490976.2020.1809331

**Published:** 2020-10-08

**Authors:** Hong-Yan Chen, Yi Hu, Nong-Hua Lu, Yin Zhu

**Affiliations:** Department of Gastroenterology, The First Affiliated Hospital of Nanchang University, Nanchang, Jiangxi Province, China

**Keywords:** Caudal type homeoboxes, intestinal metaplasia, *helicobacter pylori*, sry- related high-mobility group box 2, sonic hedgehog

## Abstract

Helicobacter pylori

(*H. pylori*), a common pathogenic bacterium in the stomach, has been demonstrated to be a major cause of gastric cancer (GC). The typical pathological evolution of *H. pylori* infection-induced GC involves development from gastric atrophy, via intestinal metaplasia (IM) and dysplasia, to intestinal-type GC. During this process, IM is considered to be an “irreversible point” that significantly increases the risk for GC. Therefore, the elucidation of the mechanism underlying IM is of great significance for the prevention and treatment of gastric mucosal carcinogenesis associated with *H. pylori* infection. Caudal type homeoboxes (CDXs) are transcription factors involved in intestinal differentiation establishment and the maintenance of normal intestinal mucosa and IM. *H. pylori* infection increases the expression of CDXs through epigenetic regulation, the nuclear factor-kappaB signaling pathway and its downstream proinflammatory factors, and the transforming growth factor-beta signaling pathway, leading to the progression from normal gastric mucosa to IM. However, the precise mechanisms of gastric intestinal metaplasia have not yet been fully elucidated. In this review, we focus on research progress revealing the functions of CDXs in *H. pylori* infection-induced IM, as well as the regulators modulating this process.

## Introduction

The most recent epidemiological data indicated that gastric cancer (GC) remains the fifth most frequently diagnosed cancer and the third leading cause of cancer-related deaths worldwide.^1^ The prognosis of patients with advanced GC remains poor and there are no efficient treatment strategies, imposing an enormous global disease and economic burden. Therefore, it is of great importance to elucidate the mechanism of gastric carcinogenesis and to develop an effective strategy for the primary prevention and treatment of GC. *H. pylori* infection is the leading pathogen causing infection-induced-cancers, accounting for 35.4% of all cases,^2^ and 90% of non-cardia GC cases have been attributed to *H. pylori* infection.^3^

*H. pylori* infection causes chronic active gastritis in all infected subjects, which can progress to gastric atrophy, intestinal metaplasia (IM), dysplasia and ultimately GC in a subset of individuals. *H. pylori* acts as an “initiation factor” during the typical pathological evolution of GC.^4^ Although timely *H. pylori* eradication can significantly reduce gastritis and reverse gastric atrophy, it has no obvious effect on IM, suggesting that the IM may be an “irreversible point” in the pathological evolution of the gastric mucosa.^5–8^Therefore, detailed investigation of the mechanism of gastric mucosal IM is of great significance for the prevention and treatment of gastric mucosal carcinogenesis associated with *H. pylori* infection.

Histologically, gastric intestinal metaplasia (GIM) involves replacement of the normal gastric epithelium with an epithelium resembling that of the intestine.^9^ The interplay of multiple risk factors has been associated with the development of GIM, including *H. pylori* infection, host genetic factors and other environmental factors (e.g., dietary habits, smoking and chronic bile acid reflux).^10–14^ CDXs are intestine-specific transcription factors regulating the intestinal epithelial cell phenotype, which are mainly present in the small intestine and colon and are rarely expressed in the normal gastric mucosa.^15^ The expression levels of CDX1 and CDX2 (CDX1/2) were shown to be significantly increased in gastric mucosal metaplastic lesions, and were closely related to *H. pylori* infection.^16^ Therefore, understanding the complex regulatory mechanisms of CDXs could offer some insights into the pathogenic mechanisms contributing to IM and ultimately GC development. In this review, we focus on the functions of CDXs in *H. pylori* infection-induced IM, as well as the regulators modulating this process, which may help to identify novel targets for treatment.

## Gastric intestinal metaplasia

Chronic mucosal inflammation eventually leads to the atrophy of multifocal glands, the loss of parietal cells, and a reduction in acid secretion. As atrophy progresses, mucin-containing goblet cells appear, representing the most prominent feature of IM.^4^ As an intermediate lesion in the development of intestinal-type GC, GIM may represent a general repair mechanism that provides a protective barrier to the epithelium in response to acute mucosal injury. However, persistent gastric injury and chronic inflammation can increase the risk of GC.

Spasmolytic polypeptide-expressing metaplasia (SPEM) is another metaplastic phenotype of gastric mucosal metaplasia caused by chronic *H. pylori* infection that was proposed based on findings in GC animal models.^17^ SPEM and goblet cell IM are recognized precancerous lesions, that are closely related to the occurrence of GC.^18^ When the gastric mucosa responds to acute injury, parietal cells are lost, and then the mature chief cells transdifferentiate to undergo SPEM, which occurs in the gastric fundus and corpus.^17,19,20^ The phenotype of SPEM is similar to that of the deep antral glands and Brunner’s glands in the duodenum with respect to the secretion of trefoil factor 2 (TFF2, also known as antispasmodic peptide) and mucin6 (MUC6),^21,22^ as a repair response initiated by the body to promote gastric mucosal healing and recovery. SPEM was the main pathological change to the gastric mucosa observed in Mongolian gerbils infected with *H. pylori*,^23^ and mixed glands expressing SPEM and IM could be observed with the extension of the infection period by at least one year.^17^ In 2004, a cohort study found that SPEM was associated with early GC in Iceland.^18^ To further clarify the relationship between SPEM and intestinal metaplasia, Goldenring et al.^24^ examined the morphological characteristics of SPEM and IM in gastric resections specimens containing SPEM and IM mixed glands, and they found that SPEM cells are located deep in the gland, and that the lineage of IM is located in the lumen of the gland. Through immunostaining for Ki67 and Muc2, they speculated that the SPEM phenotype could gradually transition to the IM phenotype, which means IM is not the only precursor/intermediate of GC.

GIM is a recognized risk factor for intestinal-type GC. The Maastricht V/Florence Consensus Report indicated that *H. pylori* eradication could not effectively reverse GIM.^25^ Once GIM is established, *H. pylori* eradication can only partially reduce the risk of GC development. GIM can be divided into complete IM and incomplete IM based on hematoxylin and eosin staining. Complete IM shows a similar histology to the small intestinal epithelium comprising mature absorptive columnar cells, goblet cells, and Paneth cells with a brush border. By contrast, the histology of incomplete IM resembles that of the colonic epithelium, with goblet cells of various sizes and mucin-secreting columnar cells with no brush border evident. Based on mucin-based classification, incomplete IM is further subdivided into type II and type III IM according to specific staining patterns, in which type I GIM corresponds to complete IM. Therefore, the histopathological classifications of complete IM and incomplete IM are usually not mutually exclusive, and occasionally mixed features of complete and incomplete forms are observed, and are defined as “mixed, mainly complete” and “mixed, mainly incomplete”.^26–28^ A technical review by the American Gastroenterological Association combined the results of seven studies reporting data on the progression of GIM to dysplasia or cancer according to histologic subtype, demonstrating that patients with incomplete GIM had a 3.3-fold higher risk of developing GC than the risk for patients with complete GIM during the 3–12.8 years of follow up.^29^ This finding suggested that incomplete IM might be of great clinical significance.^30^ However, due to the limitations of staining techniques, the lack of “best-practice” endoscopic and histological protocols for gastric preneoplasia and a relatively limited body of literature supporting the prognostic value of GIM subtyping, histological typing is still underutilized in clinical practice.^31^ The underutilization is despite wide recognition that incomplete IM is associated with a higher risk of GC. Without accurate biomarkers, it is advisable to establish GIM histological classification criteria and to perform routine histological typing for early screening to promote GC prevention.^31^

From a histological perspective, incomplete IM is accompanied by the co-expression of intestinal mucin and gastric mucin, while complete IM is associated with de novo expression of intestinal mucin, but the loss of gastric mucin expression.^32^ This difference is because, during the development of IM, there is a reduction in the gastric-specific transcription factor SRY-related high-mobility group box 2 (SOX2) and the ectopic appearance of intestinal-specific transcription factor CDXs. CDXs are the main regulatory genes for intestinal differentiation, and they play an important role in the development and maintenance of the intestinal epithelium. Much research has been conducted to explore the mechanism by which *H. pylori* regulates the ectopic expression of CDXs leading to the occurrence of GIM, and this research is reviewed in detail below.

## Role of CDXs during intestinal development

The homeobox (HOX) gene was first isolated from *Drosophila*, and encodes an evolutionarily conserved transcription factor that plays an important role in embryo growth, differentiation and development.^33^ The CDX proteins CDX1 and CDX2 belong to the ParaHox cluster of the HOX family, and they regulate the expression of intestine-specific proteins, including sucrase isomaltase,^34^ mucin2,^35^ KLF4,^36^ and LI-cadherin,^37^ to promote columnar morphogenesis and an intestinal cell phenotype. CDX1/2 show distinct expression patterns in the intestine. CDX1 expression is highest in the distal colon, whereas CDX2 expression is highest in the proximal colon; furthermore, the expression level of CDX1 was shown to gradually increase along the crypt-villus axis, and was more abundant in the crypts than in the villi, whereas CDX2 was found to be uniformly expressed along the crypt-villus axis, but was differentially phosphorylated.^16^ In addition, neither CDX1 nor CDX2 is expressed in normal gastric and esophageal tissues.^16,38^ Structurally, CDX1/2 have a high degree of homology along with overlapping functions in regulating intestinal homeostasis and colon configuration in adults.^39,40^ Several studies also showed that *CDX1* and *CDX2* exhibit transcriptional specificity in the intestine to regulate the expression of certain specific genes:^41–43^ intestinal alkaline phosphatase (an enterocyte differentiation marker gene regulated by CDX1) and the apical sodium-dependent bile acid transporter (involved in bile acid absorption and regulated by CDX1/2).

## Roles of CDXs in *H. pylori*-induced GIM

Immunohistochemical analyzes of tissue samples from patients and animal models with GIM revealed that abnormal expression patterns of CDX1/2 were observed in animals and human GIM,^44–46^ and their expression levels were closely related to the IM grade of the corpus,^38,47^ indicating that CDX1/2 may play an important role in the development of GIM. Transgenic animals confirmed the causal links between the ectopic expression of CDX1/2 and gastric epithelial transdifferentiation.^45,48^ When the gene fragment of *Cdx1/2* is inserted after the H^+^/K^+^ ATPase promoter, the parietal cells express *Cdx1/2* specifically. Therefore, CDX2 is ectopically expressed in the gastric body rather than the gastric antrum. The results showed that the gastric mucosa of *Cdx1/2* transgenic mice was replaced by intestinal epithelium after approximately 8 months. Moreover, Mutoh et al.^48^ confirmed that GIM induced by CDX1 was different from that induced by CDX2 in terms of differentiation, structure and proliferation. *H. pylori* infection is closely related to GIM formation. *H. pylori* infection can induce the expression of CDX1/2 in the gastric mucosa and their expression levels in *H. pylori*-positive patients are significantly higher than those in patients without *H. pylori* infection.^47^ *In vitro*, the expression level of CDX2 increased in GC cell lines infected by *H. pylori*, and abnormal expression of CDX1 was induced by infection with CagA-positive *H. pylori* strains.^49,50^ In addition, some studies indicated that the CDX2 expression level will decrease after *H. pylori* eradication prior to the development of GIM.^51,52^ However, a prospective study showed that *H. pylori* eradication had no significant effect on GIM development.^53^ According to these lines of evidence, it can be concluded that the high expression of CDX1/2 in the stomach is related to *H. pylori* infection and GIM, but their specific roles require further clarification.^54^

Eda et al.^55^found that CDX2 could be detected in patients with chronic gastritis and that the expression of CDX2 preceded that of CDX1 during the progression of IM. Similarly, Satoh et al.^56^also observed CDX2 expression in the gastric epithelium of *H. pylori*-infected patients, with or without obvious IM. In *Cdx2*-transgenic mouse GIM lesions, transgenic *Cdx2* induced endogenous *Cdx1* expression through the binding of *Cdx2* to the unmethylated *Cdx1* promoter region,^57^ which can potentially explain why CDX2 tends to be expressed before CDX1 and further suggests that CDX2 might act as a trigger for the development of IM.

Yeoh et al.^58^ first proposed that the CDX2 expression level progressively decreased in human GIM, dysplasia and GC, and the expression level of CDX2 in incomplete IM was found to be significantly lower than that in complete IM. Reverse transcription-polymerase chain reaction analysis of *CDX1/2* genes in 270 tissue specimens including 90 patients with GC, 90 patients with dysplasia, and 90 normal control subjects, showed that the expression level of *CDX1* in the dysplasia group was significantly higher than that in the control group, and the expression level of *CDX1/2* in the GC group was significantly higher than that in the normal control group.^47^

Wang et al.^59^carried out a meta-analysis of published literature, which first proposed that CDX2 overexpression was significantly associated with sex, a lower clinical stage, tumor differentiation, and a lower rate of vascular invasion and lymph node metastasis, as well as a higher 5-year survival rate. Analyses of publicly available databases (Kaplan-Meier plotter and The Cancer Genome Atlas) showed that GC patients with higher levels of CDX expression had significantly better clinical outcomes.^60^ *In vitro*, the overexpression of CDX2 inhibited the growth and invasion of GC cells (MKN45 cells) and reversed epithelial-to-mesenchymal transformation (EMT). *In vivo*, tumorigenicity experiments further confirmed the inhibitory effect of CDX2 on the growth and EMT of GC xenografts in nude mice^61^. The overexpression of CDX2 caused the cell cycle to stall at G0/G1, and the GC cells could not enter the next phase, thereby inhibiting their proliferation. In general, we speculate that *CDX2* may have opposing roles in different stages of gastric oncogenesis. In the early stage of *H. pylori* infection, *CDX2* acts as an oncogene to drive the transdifferentiation of the gastric epithelial phenotype into the intestinal epithelial phenotype. In the late stage of infection, the intestinalized gastric mucosa further progresses to dysplasia and GC, and *CDX2* inhibits the invasion and growth of GC as it is a tumor suppressor gene. However, further clinical studies are still needed to confirm the role of CDX2 in clinical practice and the exact prognostic significance of CDX2 in patients with GC.

## Regulation mechanism of CDXs in GIM induced by *H. pylori*

### Epigenetic regulation of CDX1/2

*H. pylori* induces chronic inflammation and IM through genetic and epigenetic changes that induce the activation of intracellular signaling pathways. Rau et al.^62^ investigated epigenetic changes in *CDXs* during the development of intestinal-type GC, revealing that the *CDX1* promoter methylation level was reduced during *H. pylori* gastritis and IM; it reached the lowest level in dysplasia. When further developed into GC, the *CDX1* promoter methylation level rose again. Moreover, NF-κB signaling activated *CDX1* expression by directly binding to its unmethylated promoter, initiating gastric mucosal transdifferentiation. Their findings suggested that the *CDX1* methylation pattern from low-grade to high-grade lesions may be a suitable predictive biomarker of GC, and *CDX2* promoter methylation did not affect its transcription. In addition to the level of methylation that affects the expression of *CDXs, CDX* autoregulation also plays an important role in CDXs. CDX2 directly activates its own transcription by binding to its own promoter, and it maintains endogenous expression in IM lesions in human and mouse intestines.^63^ CDX1 can also automatically regulate its own expression, but does not directly bind to its own promoter; instead, the complex formed by the interaction of CDX1 and LEF1 (a nuclear effector of Wnt signaling) binds to the Lef/TCF response element on the CDX1 promoter to initiate transcription.^64^ Such autoregulation of CDXs may partially explain the irreversibility of GIM; thus, interference with the autoregulation of this IM loop may be an effective strategy for GC prevention. Ectopic expression of CDX1 conferred gastric epithelial cells with an intestinal phenotype via the induction of the stemness-associated reprogramming factors SALL4 and KLF5.^65^ Relatively few studies focus on the effect of *H. pylori* infection on CDX1 expression compared to those focused on CDX2 expression. Therefore, we further discuss research progress on the detailed regulatory mechanism of CDX2 by *H. pylori.*

### NF-κB signaling pathway and pro-inflammatory cytokines

The NF-κB signaling pathway is an inflammatory-related signaling pathway that plays an important role in the progression from gastritis to GC. *H. pylori* can activate NF-κB in the gastric mucosa through Cag pathogenicity island (PAI)-dependent and CagPAI-independent pathways. As the main virulence factor of *H. pylori*, CagA translocates to the cytosol and leads to the activation of the NF-κB signaling pathway by the type IV secretion system (T4SS).^66^ During the activation of CagPAI-independent pathways, other components of *H. pylori* (e.g. vacuolating cytotoxin,^67^ peptidoglycan,^68^ urease,^69^ and outer membrane proteins^70^) can interact with corresponding receptors or targets to activate the NF-κB signaling pathway. NF-κB activation induces the release of pro-inflammatory cytokines, such as tumor necrosis factor-α, interleukin (IL)-1β, and IL-6.^71–73^ These pro-inflammatory mediators supported cell growth and proliferation by activating cellular signal transduction.^74^

IL-6 is well known to play a role as a tumorigenic factor in GC,^75–78^ and higher serum IL-6 levels are independent predictors of a poor prognosis in patients with GC.^79,80^ IL-6 binds to the alpha subunit of its specific receptor and to the gp130 homodimer on the cell membrane,^81^ and it activates two major signaling pathways: SHP-2/ERK and JAK/STAT.^82,83^ The SHP2/ERK and JAK/STAT signaling pathways are considered to play opposing roles in gastric epithelial cells, and their balance maintains the homeostasis of the gastrointestinal mucosa. *H. pylori* infection disturbs the balance between the SHP-2/ERK pathway and the JAK/STAT pathway, thereby inducing subsequent pathological changes.^84^ STAT3 promotes the growth or migration of epithelial cells and plays a role as a cancer-promoting factor in GC. Although the current view is that the SHP2/ERK signaling pathway is mainly a proliferation pathway,^85^ ERK signaling was found to play a greater role in inhibiting proliferation and inducing cell differentiation in the gastric mucosa of a *gp130.^75^,^7^,* knock-in mutant mouse model, which prevents gp130-mediated SHP2 signaling, so that the balance tilts toward the STAT3 pathway, leading to the development of GC^86^ Bolós et al.^87^ found that two signaling pathways in MKN45 cells were activated after IL-6 stimulation, which cooperatively promoted the expression of CDX2.^88^ When IL-6 stimulates the *CDX2* promoter of another GC cell line, NUGC-4, to release c-JUN, a member of the activation protein-1 transcription factor family regulating the last step of the SHP-2/ERK pathway, the c-JUN then combines with *p*-STAT3 to activate the JAK/STAT pathway to enhance CDX2 expression.^87^ These studies demonstrated that the SHP-2/ERK/MAPK signaling pathway has different regulatory effects on CDX2 in different cell types, and the specific mechanisms by which these effects occur are not completely clear.

**Figure 1.**
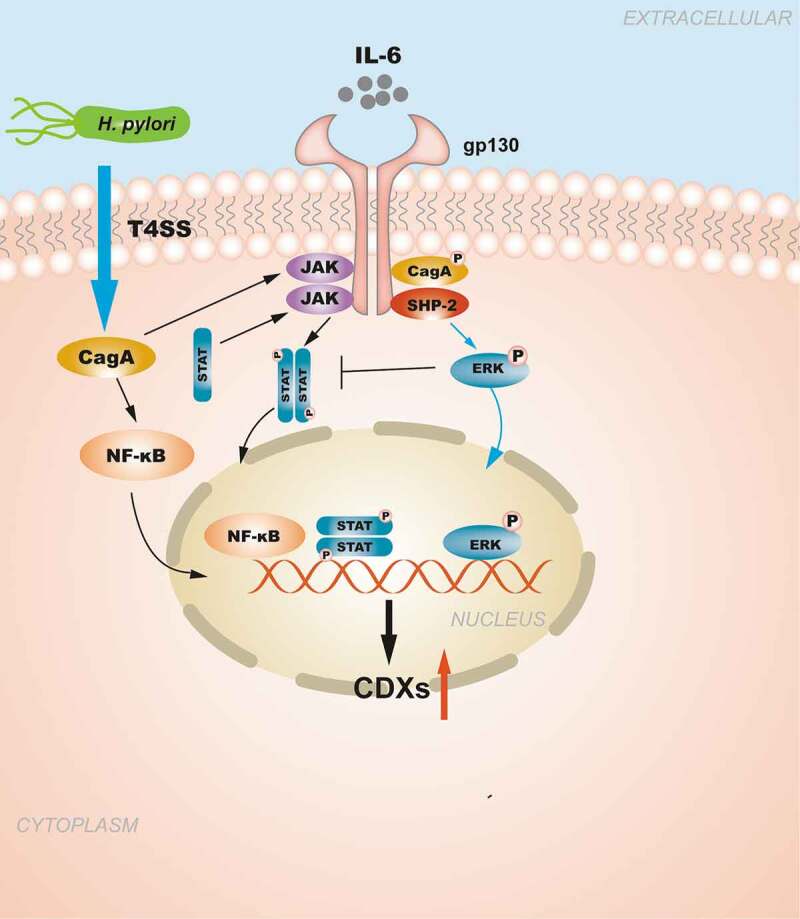
**The regulation of CDXs in GIM induced by NF-κB signaling pathway and pro-inflammatory cytokines**. *H. pylori* injects the oncoprotein CagA into gastric epithelial cells using a type IV secretion system (T4SS) to activate the NF-κB signaling pathway. NF-κB activation induces the release of the pro-inflammatory cytokine IL-6, and IL-6 binds to its specific receptor gp130, activating two major signaling pathways: SHP-2/ERK and JAK/STAT. CagA can also affect the signal transduction of gp130 regulation, and the resulting biological effect depends on the tyrosine phosphorylation status of CagA. Moreover, the SHP2/ERK and JAK/STAT signaling pathways are considered to play opposite roles in gastric epithelial cells.

In addition to IL-6 binding to human gastric mucosal gp130, the *H. pylori* virulence factor CagA can also affect the signal transduction of gp130 regulation in epithelial cells, and the resulting biological effect depends on the tyrosine phosphorylation status of CagA.^89^ Phosphorylated CagA was shown to preferentially activate the SHP2/ERK pathway, inducing cell morphological changes and cell growth inhibition, whereas non-phosphorylated CagA preferentially activates the JAK/STAT pathway to promote cell migration.^89^ In summary, the SHP2/ERK/MAPK and JAK/STAT signaling pathways may play a crucial role in CDX2 regulation and are closely related to the occurrence of GIM and GC (**Figure 1**).

*H. pylori* infection triggers the body’s innate immune response to resist further damage to the gastric mucosa, and Asano et al.^90^ found that the bacterial pattern recognition molecule nucleotide-binding oligomerization domain 1 (NOD1) could attenuate the expression of CDX2 in *H. pylori*-infected gastric epithelial cells through the activation of the NF-κB signaling pathway inhibitor TRAF3. NOD1-deficient *H. pylori*-infected mice further indicated this causal relationship based on decreasing TRAF3 expression levels, increased CDX2 expression levels, and eventual development of GIM.

### Transforming growth factor-beta (TGF-β) signaling pathway

Bone morphogenetic protein (BMP)is the largest subfamily of the TGF-β growth factor superfamily, which controls several essential biological processes, such as mesoderm formation, nervous system differentiation, dental and skeletal development, and carcinogenesis, by regulating the activity of a series of downstream genes.^91^ The BMP pathway also plays a key role in gastrointestinal development and maintaining tissue homeostasis in adults. Mutations in the BMP pathway can cause juvenile polyposis, leading to the loss of intestinal differentiation and the appearance of gastric differentiation in the intestine,^92,93^ as demonstrated in mice with mutated BMP signaling pathways.^94^ TGF-β signaling is mainly achieved by activating Smad protein. In 2008, Almeida et al.^95^ first reported that the BMP pathway was active in IM, and the key proteins in this pathway (BMP2 and BMP4) co-localized with CDX2. The same group further proved that *H. pylori* infection could activate the BMP signaling pathway, as demonstrated by the increased expression levels of BMP2, SMAD4 and pSMAD1/5/8 and increased interaction with SMAD4, to eventually upregulate CDX2 expression concomitantly with SOX2 downregulation.^95^

**Figure 2.**
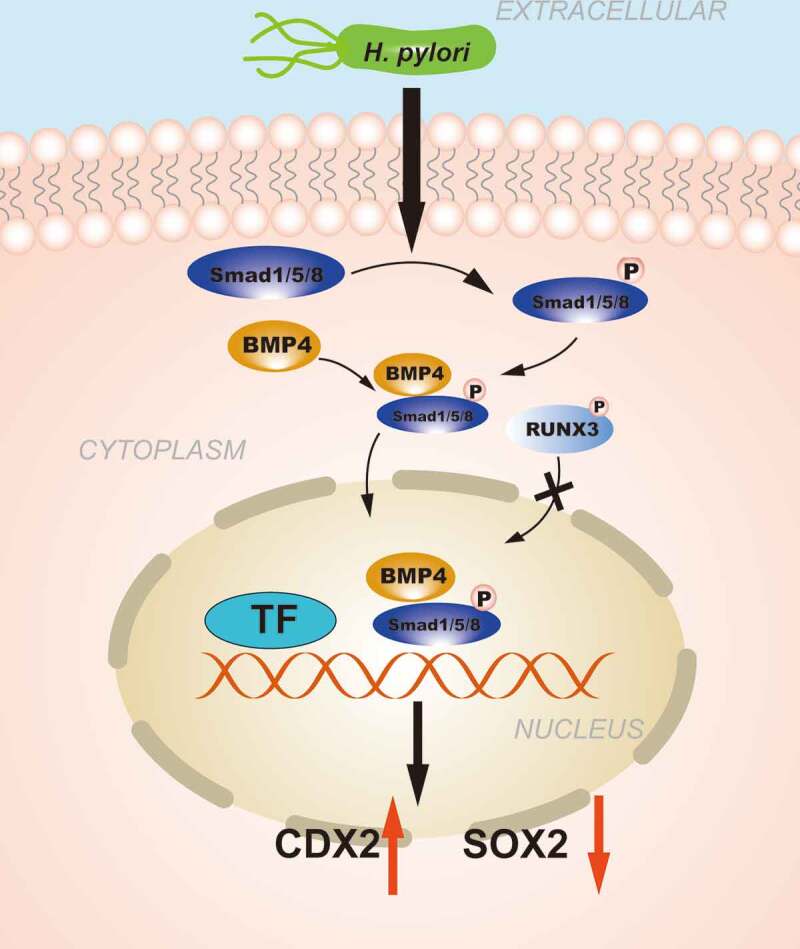
**The regulation of CDXs in GIM induced by Transforming growth factor-beta (TGF-β) signaling pathway**. *H. pylori* infection promotes the phosphorylation of SMAD1/5/8 and forms a complex with SMAD4, the complex is further transferred from the cytoplasm to the nucleus to regulate the transcription of CDX2. RUNX3 is also involved in the regulation of CDXs, abnormal cytoplasmic localization of RUNX3 caused by phosphorylation prevents it from exerting biological effects.

The Smad complex must be guided by the Runt-related transcription factor gene (RUNX) protein (including the RUNX3 protein) before it can be transferred from the cytoplasm to a specific target site. The Smad complex must co-transcribe and activate target genes with the RUNX protein, thereby affecting cell differentiation, cell cycle regulation, apoptosis and tumorigenesis. *RUNX3* is an important transcription factor downstream of the TGF-β signaling pathway as a tumor suppressor gene for GC.^96^ In the progression of GC, *RUNX3* can also undergo epigenetic changes. Low expression of RUNX3 caused by *RUNX3* hypermethylation^97^ and abnormal cytoplasmic localization of RUNX3 were observed in most IM patients with *H. pylori* infection, which is related to Src-mediated tyrosine phosphorylation, cytoplasmic RUNX3 cannot enter the nucleus and exert biological activity.^98^ Ito et al.^99^found that the gastric epithelium of *Runx3^−/-^* mice was transformed by SPEM, demonstrating MUC6 and TFF2 expression, and the expression of Cdx2 was detected in the metaplastic area. This evidence indicates that the inactivation of *RUNX3* may promote the expression of CDX2 and accelerate the formation of IM (**Figure 2**).

### CDX2 targets sonic hedgehog (Shh), instead of SOX2, to induce GIM development

SOX2 and CDX2 expression have been reported to be directly negatively correlated, suggesting that CDX2 might induce GIM by decreasing SOX2 expression.^100–102^ However, Mutoh et al.^103^ found that SOX2 expression remained stable in *Cdx2*-transgenic mice with stomach-specific expression of Cdx2. This implied that *SOX2* is not a downstream target gene of CDX2, and that ectopic CDX2 expression and low SOX2 expression in the stomach may affect GIM independently. Previous studies have shown that *H. pylori* could downregulate SOX2 expression through the IL-4/STAT6 signaling pathway. Hence, the interaction between CDX2 and SOX2 in lesions of human GIM deserves further exploration.

The loss of Shh expression has also been observed in the mucosa of *Cdx2* transgenic mice with GIM,^103^ which was proven to be caused by the binding of *Cdx2* to the unmethylated *Shh* promoter.^104^ Shh, as a morphogen, is involved in the differentiation of the gastric fundus glands in mice and human adults.^105^ Shh is secreted by the parietal cells of the gastric fundus glands to repair damage to the gastric epithelium.^106^ With the loss of parietal cells, the secretion of Shh also decreases. The gastric mucosa of *Shh*-null mice showed epithelial hyperplasia and alkaline phosphatase expression, representing a state of intestinal epithelial differentiation.^105^ In the early stages of infection, an increase in Shh expression, observed in *H. pylori* infection of gastric organoids, was regulated by activation of the NF-κB signaling.^107^ The significance of the promotion of Shh expression can be understood as the promotion of the healing of gastric mucosal injury. When inflammatory injury is further exacerbated to the stage of chronic atrophic gastritis and GIM, complete loss of parietal cells is accompanied by a loss of Shh expression.^108,109^ The eradication of *H. pylori* could improve the inflammatory status of gastric mucosa in subjects, as evidenced by an increase in serum pepsinogen I levels along with an increase in Shh expression and a decrease in CDX2 expression.^52^ Collectively, this evidence indicates that in addition to activating the de novo expression of intestinal mucin, CDX2 can directly downregulate Shh expression to weaken the repair function of the gastric epithelium, and low levels of SOX2 reduce the secretion of gastric mucin to play a synergistic role, leading to the transformation of the normal gastric mucosa into the IM phenotype.

## Conclusions and Future Perspectives

**Figure 3.**
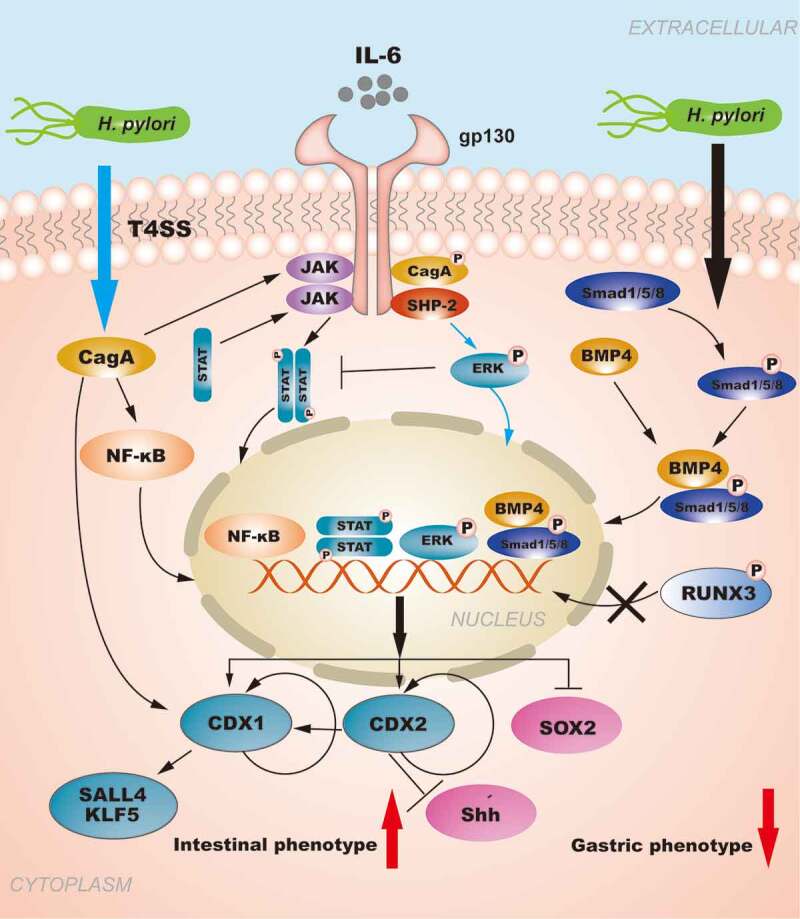
**The role of CDXs in GIM: The regulation of CDXs in GIM induced by *H. pylori***. *H. pylori* injects the oncoprotein CagA into gastric epithelial cells using a type IV secretion system (T4SS) to activate the NF-κB signaling pathway. NF-κB activation induces the release of the pro-inflammatory cytokine IL-6, and IL-6 binds to its specific receptor gp130, activating two major signaling pathways: SHP-2/ERK and JAK/STAT. CagA can also affect the signal transduction of gp130 regulation, and the resulting biological effect depends on the tyrosine phosphorylation status of CagA. Moreover, the SHP2/ERK and JAK/STAT signaling pathways are considered to play opposite roles in gastric epithelial cells, and SHP-2/ERK signaling plays a greater role in inhibiting proliferation and inducing cell differentiation in the gastric mucosa. *H. pylori* infection can activate the BMP pathway to upregulate CDX2 expression concomitantly with SOX2 downregulation. RUNX3 is also involved in the regulation of CDXs. Furthermore, the autoregulation of CDXs maintains their expression in GIM. In summary, *H. pylori* infection is responsible for increasing the levels of intestinal-specific transcription factors and decreasing the levels gastric-specific transcription factors, which contribute to the development of GIM.

Early eradication of *H. pylori* can provide a fundamental solution to prevent GC caused by *H. pylori* infection. However, when gastric mucosal lesions progress to IM, *H. pylori* eradication does not effectively prevent the transition to tumor development. *H. pylori* infection is responsible for increasing the levels of intestinal-specific transcription factors and decreasing gastric-specific transcription factors, which contribute to the development of GIM (**Figure 3**). The metaplastic expression of CDXs in the stomach is closely related to inflammatory stimuli. Therefore, the elucidation of the regulatory mechanism of CDXs in GIM caused by *H. pylori* infection will contribute to the development of further targeted therapies for early intervention and treatment strategies for GC. Simultaneously, the establishment and dissemination of GIM endoscopic and histological protocols along with histological typing criteria are urgently needed to improve risk stratification for GC.

## References

[1] Bray F, Ferlay J, Soerjomataram I, Siegel RL, Torre LA, Jemal A. Global cancer statistics 2018: GLOBOCAN estimates of incidence and mortality worldwide for 36 cancers in 185 countries.. CA: A Cancer Journal for Clinicians. 2018;68(6):394–424. doi:10.3322/caac.21492. 30207593

[2] Plummer M, de Martel C, Vignat J, Ferlay J, Bray F, Franceschi S. Global burden of cancers attributable to infections in 2012: a synthetic analysis. Lancet Glob Health. 2016;4:e609–16. 27470177 10.1016/S2214-109X(16)30143-7

[3] Chen W, Zheng R, Baade PD, Zhang S, Zeng H, Bray F, Jemal A, Yu XQ, He J. Cancer statistics in China, 2015. CA Cancer J Clin. 2016;66:115–132. 26808342 10.3322/caac.21338

[4] Correa P. Human gastric carcinogenesis: a multistep and multifactorial process–First American Cancer Society Award Lecture on Cancer Epidemiology and Prevention. Cancer Res. 1992;52:6735–6740. 1458460

[5] Lee YC, Chen TH, Chiu HM, Shun CT, Chiang H, Liu TY, Liu TY, Wu MS, Lin JT. The benefit of mass eradication of Helicobacter pylori infection: a community-based study of gastric cancer prevention. Gut. 2013;62:676–682. 22698649 10.1136/gutjnl-2012-302240 PMC3618687

[6] Chen HN, Wang Z, Li X, Zhou ZG. Helicobacter pylori eradication cannot reduce the risk of gastric cancer in patients with intestinal metaplasia and dysplasia: evidence from a meta-analysis. Gastric Cancer. 2016;19:166–175. 25609452 10.1007/s10120-015-0462-7

[7] Wang J, Xu L, Shi R, Huang X, Li SW, Huang Z, Zhang G. Gastric atrophy and intestinal metaplasia before and after Helicobacter pylori eradication: a meta-analysis. Digestion. 2011;83:253–260. 21282951 10.1159/000280318

[8] Rokkas T, Pistiolas D, Sechopoulos P, Robotis I, Margantinis G. The long-term impact of Helicobacter pylori eradication on gastric histology: a systematic review and meta-analysis. Helicobacter. 2007;12:32–38. 17991174 10.1111/j.1523-5378.2007.00563.x

[9] Park YH, Kim N. Review of atrophic gastritis and intestinal metaplasia as a premalignant lesion of gastric cancer. J Cancer Prev. 2015;20:25–40. 25853101 10.15430/JCP.2015.20.1.25 PMC4384712

[10] Correa P, Houghton J. Carcinogenesis of Helicobacter pylori. Gastroenterology. 2007;133(2):659–672. doi:10.1053/j.gastro.2007.06.026. 17681184

[11] Song JH, Kim YS, Heo NJ, Lim JH, Yang SY, Chung GE, et al. High Salt Intake Is Associated with Atrophic Gastritis with Intestinal Metaplasia. Cancer Epidemiol Biomarkers Prev. 2017;26:1133–1138. 28341758 10.1158/1055-9965.EPI-16-1024

[12] Kneller RW, You WC, Chang YS, Liu WD, Zhang L, Zhao L, et al. Cigarette smoking and other risk factors for progression of precancerous stomach lesions. J Natl Cancer Inst. 1992;84:1261–1266. 1640486 10.1093/jnci/84.16.1261

[13] Leung WK, Lin SR, Ching JY, To KF, Ng EK, Chan FK, et al. Factors predicting progression of gastric intestinal metaplasia: results of a randomised trial on Helicobacter pylori eradication. Gut. 2004;53:1244–1249. 15306578 10.1136/gut.2003.034629 PMC1774213

[14] Matsuhisa T, Arakawa T, Watanabe T, Tokutomi T, Sakurai K, Okamura S, et al. Relation between bile acid reflux into the stomach and the risk of atrophic gastritis and intestinal metaplasia: a multicenter study of 2283 cases. Dig Endosc. 2013;25:519–525. 23363381 10.1111/den.12030

[15] Silberg DG, Swain GP, Suh ER, Traber PG. Cdx1 and cdx2 expression during intestinal development. Gastroenterology. 2000;119:961–971. 11040183 10.1053/gast.2000.18142

[16] Guo RJ, Suh ER, Lynch JP. The role of Cdx proteins in intestinal development and cancer. Cancer Biol Ther. 2004;3:593–601. 15136761 10.4161/cbt.3.7.913

[17] Yoshizawa N, Takenaka Y, Yamaguchi H, Tetsuya T, Tanaka H, Tatematsu M, et al. Emergence of spasmolytic polypeptide-expressing metaplasia in Mongolian gerbils infected with Helicobacter pylori. Lab Invest. 2007;87:1265–1276. 18004396 10.1038/labinvest.3700682

[18] Halldorsdottir AM, Sigurdardottrir M, Jonasson JG, Oddsdottir M, Magnusson J, Lee JR, et al. Spasmolytic polypeptide-expressing metaplasia (SPEM) associated with gastric cancer in Iceland. Dig Dis Sci. 2003;48:431–441. 12757153 10.1023/a:1022564027468

[19] Nam KT, Lee HJ, Sousa JF, Weis VG, O’Neal RL, Finke PE, et al. Mature chief cells are cryptic progenitors for metaplasia in the stomach. Gastroenterology. 2010;139:2028–37 e9. 20854822 10.1053/j.gastro.2010.09.005 PMC2997152

[20] Weis VG, Sousa JF, LaFleur BJ, Nam KT, Weis JA, Finke PE, et al. Heterogeneity in mouse spasmolytic polypeptide-expressing metaplasia lineages identifies markers of metaplastic progression. Gut. 2013;62:1270–1279. 22773549 10.1136/gutjnl-2012-302401 PMC3762676

[21] Weis VG, Goldenring JR. Current understanding of SPEM and its standing in the preneoplastic process. Gastric Cancer. 2009;12:189–197. 20047123 10.1007/s10120-009-0527-6 PMC4502916

[22] Goldenring JR, Nam KT, Mills JC. The origin of pre-neoplastic metaplasia in the stomach: chief cells emerge from the Mist. Exp Cell Res. 2011;317:2759–2764. 21907708 10.1016/j.yexcr.2011.08.017 PMC3210373

[23] Shimizu T, Choi E, Petersen CP, Noto JM, Romero-Gallo J, Piazuelo MB, Washington MK, Peek RM, Jr., Goldenring JR. Characterization of progressive metaplasia in the gastric corpus mucosa of Mongolian gerbils infected with Helicobacter pylori. J Pathol. 2016;239:399–410. 27125972 10.1002/path.4735 PMC4958595

[24] Goldenring JR, Nam KT, Wang TC, Mills JC, Wright NA. Spasmolytic polypeptide-expressing metaplasia and intestinal metaplasia: time for reevaluation of metaplasias and the origins of gastric cancer. Gastroenterology. 2010;138:2207-2210, 2210 e2201. 10.1053/j.gastro.2010.04.023 PMC3769643 20450866

[25] Malfertheiner P, Megraud F, O’Morain CA, Gisbert JP, Kuipers EJ, Axon AT, Bazzoli F, Gasbarrini A, Atherton J, Graham DY, et al. Management of Helicobacter pylori infection-the Maastricht V/Florence Consensus Report. Gut. 2017;66:6–30. 27707777 10.1136/gutjnl-2016-312288

[26] Correa P, Piazuelo MB, Wilson KT. Pathology of gastric intestinal metaplasia: clinical implications. Am J Gastroenterol. 2010;105:493–498. 20203636 10.1038/ajg.2009.728 PMC2895407

[27] Jass JR, Filipe MI. A variant of intestinal metaplasia associated with gastric carcinoma: a histochemical study. Histopathology. 1979;3:191–199. 468122 10.1111/j.1365-2559.1979.tb02996.x

[28] Cassaro M, Rugge M, Gutierrez O, Leandro G, Graham DY, Genta RM. Topographic patterns of intestinal metaplasia and gastric cancer. Am J Gastroenterol. 2000;95:1431–1438. 10894575 10.1111/j.1572-0241.2000.02074.x

[29] Gawron AJ, Shah SC, Altayar O, Davitkov P, Morgan D, Turner K, et al. AGA Technical Review on Gastric Intestinal Metaplasia-Natural History and Clinical Outcomes. Gastroenterology. 2020;158:705–31 e5. 31816300 10.1053/j.gastro.2019.12.001 PMC7375032

[30] Gonzalez CA, Sanz-Anquela JM, Gisbert JP, Correa P. Utility of subtyping intestinal metaplasia as marker of gastric cancer risk. A Review of the Evidence. Int J Cancer. 2013;133:1023–1032. 23280711 10.1002/ijc.28003 PMC3732516

[31] Shah SC, Gawron AJ, Mustafa RA, Piazuelo MB. Histologic Subtyping of Gastric Intestinal Metaplasia: overview and Considerations for Clinical Practice. Gastroenterology. 2020;158:745–750. 31887261 10.1053/j.gastro.2019.12.004 PMC7302270

[32] Reis CA, David L, Correa P, Carneiro F, de Bolos C, Garcia E, et al. Intestinal metaplasia of human stomach displays distinct patterns of mucin (MUC1, MUC2, MUC5AC, and MUC6) expression. Cancer Res. 1999;59:1003–1007. 10070955

[33] Beck F. Homeobox genes in gut development. Gut. 2002;51:450–454. 12171973 10.1136/gut.51.3.450 PMC1773336

[34] Tung J, Markowitz AJ, Silberg DG, Traber PG. Developmental expression of SI is regulated in transgenic mice by an evolutionarily conserved promoter. Am J Physiol. 1997;273:G83–92. 9252513 10.1152/ajpgi.1997.273.1.G83

[35] Yamamoto H, Bai YQ, Yuasa Y. Homeodomain protein CDX2 regulates goblet-specific MUC2 gene expression. Biochem Biophys Res Commun. 2003;300:813–818. 12559945 10.1016/s0006-291x(02)02935-2

[36] Dang DT, Mahatan CS, Dang LH, Agboola IA, Yang VW. Expression of the gut-enriched Kruppel-like factor (Kruppel-like factor 4) gene in the human colon cancer cell line RKO is dependent on CDX2. Oncogene. 2001;20:4884–4890. 11521200 10.1038/sj.onc.1204645 PMC2268091

[37] Hinoi T, Lucas PC, Kuick R, Hanash S, Cho KR, Fearon ER. CDX2 regulates liver intestine-cadherin expression in normal and malignant colon epithelium and intestinal metaplasia. Gastroenterology. 2002;123:1565–1577. 12404231 10.1053/gast.2002.36598

[38] Mizoshita T, Inada K, Tsukamoto T, Kodera Y, Yamamura Y, Hirai T, Kato T, Joh T, Itoh M, Tatematsu M. Expression of Cdx1 and Cdx2 mRNAs and relevance of this expression to differentiation in human gastrointestinal mucosa–with special emphasis on participation in intestinal metaplasia of the human stomach. Gastric Cancer. 2001;4:185–191. 11846061 10.1007/pl00011741

[39] Verzi MP, Shin H, Ho LL, Liu XS, Shivdasani RA. Essential and redundant functions of caudal family proteins in activating adult intestinal genes. Mol Cell Biol. 2011;31:2026–2039. 21402776 10.1128/MCB.01250-10 PMC3133364

[40] Hryniuk A, Grainger S, Savory JG, Lohnes D. Cdx function is required for maintenance of intestinal identity in the adult. Dev Biol. 2012;363:426–437. 22285812 10.1016/j.ydbio.2012.01.010

[41] Grainger S, Hryniuk A, Lohnes D. Cdx1 and Cdx2 exhibit transcriptional specificity in the intestine. PLoS One. 2013;8:e54757. 23382958 10.1371/journal.pone.0054757 PMC3559873

[42] Alkhoury F, Malo MS, Mozumder M, Mostafa G, Hodin RA. Differential regulation of intestinal alkaline phosphatase gene expression by Cdx1 and Cdx2. Am J Physiol Gastrointest Liver Physiol. 2005;289:G285–90. 15774940 10.1152/ajpgi.00037.2005

[43] Ma L, Juttner M, Kullak-Ublick GA, Eloranta JJ. Regulation of the gene encoding the intestinal bile acid transporter ASBT by the caudal-type homeobox proteins CDX1 and CDX2. Am J Physiol Gastrointest Liver Physiol. 2012;302:G123–33. 22016432 10.1152/ajpgi.00102.2011

[44] Almeida R, Silva E, Santos-Silva F, Silberg DG, Wang J, De Bolos C, David L. Expression of intestine-specific transcription factors, CDX1 and CDX2, in intestinal metaplasia and gastric carcinomas. J Pathol. 2003;199:36–40. 12474224 10.1002/path.1246

[45] Silberg DG, Sullivan J, Kang E, Swain GP, Moffett J, Sund NJ,Sackett SD, Kaestner KH. Cdx2 ectopic expression induces gastric intestinal metaplasia in transgenic mice. Gastroenterology. 2002;122:689–696. 11875002 10.1053/gast.2002.31902

[46] Silberg DG, Furth EE, Taylor JK, Schuck T, Chiou T, Traber PG. CDX1 protein expression in normal, metaplastic, and neoplastic human alimentary tract epithelium. Gastroenterology. 1997;113:478–486. 9247467 10.1053/gast.1997.v113.pm9247467

[47] Kang JM, Lee BH, Kim N, Lee HS, Lee HE, Park JH, Kim JS, Jung HC, Song IS. CDX1 and CDX2 expression in intestinal metaplasia, dysplasia and gastric cancer. J Korean Med Sci. 2011;26:647–653. 21532856 10.3346/jkms.2011.26.5.647 PMC3082117

[48] Mutoh H, Sakurai S, Satoh K, Osawa H, Hakamata Y, Takeuchi T, Sugano K. Cdx1 induced intestinal metaplasia in the transgenic mouse stomach: comparative study with Cdx2 transgenic mice. Gut. 2004;53:1416–1423. 15361487 10.1136/gut.2003.032482 PMC1774241

[49] Murata-Kamiya N, Kurashima Y, Teishikata Y, Yamahashi Y, Saito Y, Higashi H, et al. Helicobacter pylori CagA interacts with E-cadherin and deregulates the beta-catenin signal that promotes intestinal transdifferentiation in gastric epithelial cells. Oncogene. 2007;26:4617–4626. 17237808 10.1038/sj.onc.1210251

[50] Choi SI, Yoon C, Park MR, Lee D, Kook MC, Lin JX, et al. CDX1 Expression Induced by CagA-Expressing Helicobacter pylori Promotes Gastric Tumorigenesis. Mol Cancer Res. 2019;17:2169–2183. 31416838 10.1158/1541-7786.MCR-19-0181

[51] Vauhkonen M, Vauhkonen H, Sipponen P. Helicobacter pylori infection induces a reversible expression of the CDX2 transcription factor protein in human gastric epithelium. Scand J Gastroenterol. 2008;43:915–921. 19086163 10.1080/00365520802014841

[52] Shiotani A, Uedo N, Iishi H, Tatsuta M, Ishiguro S, Nakae Y,Kamada T, Haruma K, Merchant JL. Re-expression of sonic hedgehog and reduction of CDX2 after Helicobacter pylori eradication prior to incomplete intestinal metaplasia. Int J Cancer. 2007;121:1182–1189. 17520681 10.1002/ijc.22835

[53] Shin CM, Kim N, Chang H, Kim JS, Lee DH, Jung HC. Follow-Up Study on CDX1 and CDX2 mRNA Expression in Noncancerous Gastric Mucosae After Helicobacter pylori Eradication. Dig Dis Sci. 2016;61:1051–1059. 26841784 10.1007/s10620-016-4048-y

[54] Barros R, Freund JN, David L, Almeida R. Gastric intestinal metaplasia revisited: function and regulation of CDX2. Trends Mol Med. 2012;18:555–563. 22871898 10.1016/j.molmed.2012.07.006

[55] Eda A, Osawa H, Yanaka I, Satoh K, Mutoh H, Kihira K, Sugano K. Expression of homeobox gene CDX2 precedes that of CDX1 during the progression of intestinal metaplasia. J Gastroenterol. 2002;37:94–100. 11871772 10.1007/s005350200002

[56] Satoh K, Mutoh H, Eda A, Yanaka I, Osawa H, Honda S, Kawata H, Kihira K, Sugano K. Aberrant expression of CDX2 in the gastric mucosa with and without intestinal metaplasia: effect of eradication of Helicobacter pylori. Helicobacter. 2002;7:192–198. 12047325 10.1046/j.1523-5378.2002.00080.x

[57] Mutoh H, Hayakawa H, Sakamoto H, Sashikawa M, Sugano K. Transgenic Cdx2 induces endogenous Cdx1 in intestinal metaplasia of Cdx2-transgenic mouse stomach. Febs J. 2009;276:5821–5831. 19725873 10.1111/j.1742-4658.2009.07263.x

[58] Liu Q, Teh M, Ito K, Shah N, Ito Y, Yeoh KG. CDX2 expression is progressively decreased in human gastric intestinal metaplasia, dysplasia and cancer. Mod Pathol. 2007;20:1286–1297. 17906616 10.1038/modpathol.3800968

[59] Wang XT, Wei WY, Kong FB, Lian C, Luo W, Xiao Q, Xie YB. Prognostic significance of Cdx2 immunohistochemical expression in gastric cancer: a meta-analysis of published literatures. Journal of experimental & clinical cancer research: CR 2012; 31:98. 23181722 10.1186/1756-9966-31-98 PMC3533813

[60] Nakayama C, Yamamichi N, Tomida S, Takahashi Y, Kageyama-Yahara N, Sakurai K, Takeuchi C, Inada KI, Shiogama K, Nagae G. Transduced caudal-type homeobox (CDX) 2/CDX1 can induce growth inhibition on CDX-deficient gastric cancer by rapid intestinal differentiation. Cancer Sci. 2018;109:3853–3864. 30289576 10.1111/cas.13821 PMC6272106

[61] Zhang JF, Qu LS, Qian XF, Xia BL, Mao ZB, Chen WC. Nuclear transcription factor CDX2 inhibits gastric cancer‑cell growth and reverses epithelial‑to‑mesenchymal transition in vitro and in vivo. Molecular Medicine Reports. 2015;12:5231–5238. 26238762 10.3892/mmr.2015.4114

[62] Rau TT, Rogler A, Frischauf M, Jung A, Konturek PC, Dimmler A, Faller G, Sehnert B, El-Rifai W, Hartmann A, et al. Methylation-dependent activation of CDX1 through NF-kappaB: a link from inflammation to intestinal metaplasia in the human stomach. Am J Pathol. 2012;181:487–498. 22749770 10.1016/j.ajpath.2012.04.028 PMC3409443

[63] Barros R, da Costa LT, Pinto-de-Sousa J, Duluc I, Freund JN, David L, David L, Almeida R. CDX2 autoregulation in human intestinal metaplasia of the stomach: impact on the stability of the phenotype. Gut. 2011;60:290–298. 21148572 10.1136/gut.2010.222323 PMC3034084

[64] Beland M, Pilon N, Houle M, Oh K, Sylvestre JR, Prinos P, Lohnes D. Cdx1 autoregulation is governed by a novel Cdx1-LEF1 transcription complex. Mol Cell Biol. 2004;24:5028–5038. 15143193 10.1128/MCB.24.11.5028-5038.2004 PMC416402

[65] Fujii Y, Yoshihashi K, Suzuki H, Tsutsumi S, Mutoh H, Maeda S, Yamagata Y, Seto Y, Aburatani H, Hatakeyama M. CDX1 confers intestinal phenotype on gastric epithelial cells via induction of stemness-associated reprogramming factors SALL4 and KLF5. Proc Natl Acad Sci U S A. 2012;109:20584–20589. 23112162 10.1073/pnas.1208651109 PMC3528493

[66] Suzuki M, Mimuro H, Kiga K, Fukumatsu M, Ishijima N, Morikawa H, Nagai S, Koyasu S, Gilman RH, Kersulyte D, et al. Helicobacter pylori CagA phosphorylation-independent function in epithelial proliferation and inflammation. Cell Host Microbe. 2009;5:23–34. 19154985 10.1016/j.chom.2008.11.010

[67] Takeshima E, Tomimori K, Takamatsu R, Ishikawa C, Kinjo F, Hirayama T, Fujita J, Mori N. Helicobacter pylori VacA activates NF-kappaB in T cells via the classical but not alternative pathway. Helicobacter. 2009;14:271–279. 19674131 10.1111/j.1523-5378.2009.00683.x

[68] Allison CC, Kufer TA, Kremmer E, Kaparakis M, Ferrero RL. Helicobacter pylori induces MAPK phosphorylation and AP-1 activation via a NOD1-dependent mechanism. J Immunol. 2009;183:8099–8109. 20007577 10.4049/jimmunol.0900664

[69] Beswick EJ, Pinchuk IV, Minch K, Suarez G, Sierra JC, Yamaoka Y, Reyes VE. The Helicobacter pylori urease B subunit binds to CD74 on gastric epithelial cells and induces NF-kappaB activation and interleukin-8 production. Infect Immun. 2006;74:1148–1155. 16428763 10.1128/IAI.74.2.1148-1155.2006 PMC1360328

[70] Belogolova E, Bauer B, Pompaiah M, Asakura H, Brinkman V, Ertl C, Bartfeld S, Nechitaylo TY, Haas R, Machuy N, et al. Helicobacter pylori outer membrane protein HopQ identified as a novel T4SS-associated virulence factor. Cell Microbiol. 2013;15:1896–1912. 23782461 10.1111/cmi.12158 PMC3797234

[71] Fan XG, Chua A, Fan XJ, Keeling PW. Increased gastric production of interleukin-8 and tumour necrosis factor in patients with Helicobacter pylori infection. J Clin Pathol. 1995;48:133–136. 7745112 10.1136/jcp.48.2.133 PMC502381

[72] Yamaoka Y, Kita M, Kodama T, Sawai N, Kashima K, Imanishi J. Induction of various cytokines and development of severe mucosal inflammation by cagA gene positive Helicobacter pylori strains. Gut. 1997;41:442–451. 9391240 10.1136/gut.41.4.442 PMC1891528

[73] Basso D, Scrigner M, Toma A, Navaglia F, Di Mario F, Rugge M, Plebani M. Helicobacter pylori infection enhances mucosal interleukin-1 beta, interleukin-6, and the soluble receptor of interleukin-2. Int J Clin Lab Res. 1996;26:207–210. 8905454 10.1007/BF02592984

[74] Fujiki H, Sueoka E, Suganuma M. Tumor promoters: from chemicals to inflammatory proteins. J Cancer Res Clin Oncol. 2013;139:1603–1614. 23756937 10.1007/s00432-013-1455-8 PMC11824212

[75] Hong DS, Angelo LS, Kurzrock R. Interleukin-6 and its receptor in cancer: implications for translational therapeutics. Cancer. 2007;110:1911–1928. 17849470 10.1002/cncr.22999

[76] Lin MT, Lin BR, Chang CC, Chu CY, Su HJ, Chen ST, Jeng YM, Kuo ML. IL-6 induces AGS gastric cancer cell invasion via activation of the c-Src/RhoA/ROCK signaling pathway. Int J Cancer. 2007;120:2600–2608. 17304514 10.1002/ijc.22599

[77] Yadav A, Kumar B, Datta J, Teknos TN, Kumar P. IL-6 promotes head and neck tumor metastasis by inducing epithelial-mesenchymal transition via the JAK-STAT3-SNAIL signaling pathway. Mol Cancer Res. 2011;9:1658–1667. 21976712 10.1158/1541-7786.MCR-11-0271 PMC3243808

[78] Kinoshita H, Hirata Y, Nakagawa H, Sakamoto K, Hayakawa Y, Takahashi R, Nakata W, Sakitani K, Serizawa T, Hikiba Y et al. Interleukin-6 mediates epithelial-stromal interactions and promotes gastric tumorigenesis. PLoS One. 2013;8:e60914. 23593346 10.1371/journal.pone.0060914 PMC3625204

[79] Liao WC, Lin JT, Wu CY, Huang SP, Lin MT, Wu AS, Huang YJ, Wu MS. Serum interleukin-6 level but not genotype predicts survival after resection in stages II and III gastric carcinoma. Clin Cancer Res. 2008;14:428–434. 18198221 10.1158/1078-0432.CCR-07-1032

[80] Ashizawa T, Okada R, Suzuki Y, Takagi M, Yamazaki T, Sumi T, Aoki T, Ohnuma S, Aoki T. Clinical significance of interleukin-6 (IL-6) in the spread of gastric cancer: role of IL-6 as a prognostic factor. Gastric Cancer. 2005;8:124–131. 15864720 10.1007/s10120-005-0315-x

[81] Heinrich PC, Behrmann I, Muller-Newen G, Schaper F, Graeve L. Interleukin-6-type cytokine signalling through the gp130/Jak/STAT pathway. Biochem J. 1998;334:297–314. 9716487 10.1042/bj3340297 PMC1219691

[82] Heinrich PC, Behrmann I, Haan S, Hermanns HM, Muller-Newen G, Schaper F. Principles of interleukin (IL)-6-type cytokine signalling and its regulation. Biochem J. 2003;374:1–20. 12773095 10.1042/BJ20030407 PMC1223585

[83] Kamimura D, Ishihara K, Hirano T. IL-6 signal transduction and its physiological roles: the signal orchestration model. Rev Physiol Biochem Pharmacol. 2003;149:1–38. 12687404 10.1007/s10254-003-0012-2

[84] Jackson CB, Judd LM, Menheniott TR, Kronborg I, Dow C, Yeomans ND, Boussioutas A, Robb L, Giraud AS. Augmented gp130-mediated cytokine signalling accompanies human gastric cancer progression. J Pathol. 2007;213:140–151. 17724739 10.1002/path.2218

[85] Guo YJ, Pan WW, Liu SB, Shen ZF, Xu Y, Hu LL. ERK/MAPK signalling pathway and tumorigenesis. Exp Ther Med. 2020;19:1997–2007. 32104259 10.3892/etm.2020.8454 PMC7027163

[86] Tebbutt NC, Giraud AS, Inglese M, Jenkins B, Waring P, FJ C, Malki S, Alderman BM, Grail D, Hollande F, et al. Reciprocal regulation of gastrointestinal homeostasis by SHP2 and STAT-mediated trefoil gene activation in gp130 mutant mice. Nat Med. 2002;8:1089–1097. 12219085 10.1038/nm763

[87] Cobler L, Pera M, Garrido M, Iglesias M, de Bolos C. CDX2 can be regulated through the signalling pathways activated by IL-6 in gastric cells. Biochim Biophys Acta. 2014;1839:785–792. 24953186 10.1016/j.bbagrm.2014.06.009

[88] Suzuki T, Yoshinaga N, Tanabe S. Interleukin-6 (IL-6) regulates claudin-2 expression and tight junction permeability in intestinal epithelium. J Biol Chem. 2011;286:31263–31271. 21771795 10.1074/jbc.M111.238147 PMC3173073

[89] Lee IO, Kim JH, Choi YJ, Pillinger MH, Kim SY, Blaser MJ, Lee YC. Helicobacter pylori CagA phosphorylation status determines the gp130-activated SHP2/ERK and JAK/STAT signal transduction pathways in gastric epithelial cells. J Biol Chem. 2010;285:16042–16050. 20348091 10.1074/jbc.M110.111054 PMC2871473

[90] Asano N, Imatani A, Watanabe T, Fushiya J, Kondo Y, Jin X, Ara N, Uno K, Iijima K, Koike T, et al. Cdx2 Expression and Intestinal Metaplasia Induced by H. Pylori Infection of Gastric Cells Is Regulated by NOD1-Mediated Innate Immune Responses. Cancer Res. 2016;76:1135–1145. 26759244 10.1158/0008-5472.CAN-15-2272 PMC4799656

[91] von Bubnoff A, Cho KW. Intracellular BMP signaling regulation in vertebrates: pathway or network? Dev Biol. 2001;239:1–14. 11784015 10.1006/dbio.2001.0388

[92] Barros R, Mendes N, Howe JR, Reis CA, de Bolos C, Carneiro F, David L, Almeida R. Juvenile polyps have gastric differentiation with MUC5AC expression and downregulation of CDX2 and SMAD4. Histochem Cell Biol. 2009;131:765–772. 19266212 10.1007/s00418-009-0579-z

[93] Sayed MG, Ahmed AF, Ringold JR, Anderson ME, Bair JL, Mitros FA, Lynch HT, Tinley ST, Petersen GM, Giardiello FM, et al. Germline SMAD4 or BMPR1A mutations and phenotype of juvenile polyposis. Ann Surg Oncol. 2002;9:901–906. 12417513 10.1007/BF02557528

[94] Haramis AP, Begthel H, van den Born M, van Es J, Jonkheer S, Offerhaus GJ, Clevers H. De novo crypt formation and juvenile polyposis on BMP inhibition in mouse intestine. Science. 2004;303:1684–1686. 15017003 10.1126/science.1093587

[95] Barros R, Pereira B, Duluc I, Azevedo M, Mendes N, Camilo V, Jacobs RJ, Paulo P, Santos-Silva F, van Seuningen I, et al. Key elements of the BMP/SMAD pathway co-localize with CDX2 in intestinal metaplasia and regulate CDX2 expression in human gastric cell lines. J Pathol. 2008;215:411–420. 18498120 10.1002/path.2369

[96] Li QL, Ito K, Sakakura C, Fukamachi H, Inoue K, Chi XZ, Lee KY, Nomura S, Lee CW, Han SB, et al. Causal relationship between the loss of RUNX3 expression and gastric cancer. Cell. 2002;109:113–124. 11955451 10.1016/s0092-8674(02)00690-6

[97] Lu XX, Yu JL, Ying LS, Han J, Wang S, Yu QM, Wang XB, Fang XH, Ling ZQ. Stepwise cumulation of RUNX3 methylation mediated by Helicobacter pylori infection contributes to gastric carcinoma progression. Cancer. 2012;118:5507–5517. 22576578 10.1002/cncr.27604

[98] Cinghu S, Goh YM, Oh BC, Lee YS, Lee OJ, Devaraj H, et al. Phosphorylation of the gastric tumor suppressor RUNX3 following H. Pylori Infection Results in Its Localization to the Cytoplasm. J Cell Physiol. 2012;227:1071–1080. 21567391 10.1002/jcp.22820

[99] Ito K, Chuang LS, Ito T, Chang TL, Fukamachi H, Salto-Tellez M, et al. Loss of Runx3 is a key event in inducing precancerous state of the stomach. Gastroenterology. 2011;140:1536–46 e8. 21277301 10.1053/j.gastro.2011.01.043

[100] Camilo V, Barros R, Sousa S, Magalhaes AM, Lopes T, Mario Santos A, et al. Helicobacter pylori and the BMP pathway regulate CDX2 and SOX2 expression in gastric cells. Carcinogenesis. 2012;33:1985–1992. 22791809 10.1093/carcin/bgs233

[101] Asonuma S, Imatani A, Asano N, Oikawa T, Konishi H, Iijima K, et al. Helicobacter pylori induces gastric mucosal intestinal metaplasia through the inhibition of interleukin-4-mediated HMG box protein Sox2 expression. Am J Physiol Gastrointest Liver Physiol. 2009;297:G312–22. 19520737 10.1152/ajpgi.00518.2007

[102] Benahmed F, Gross I, Gaunt SJ, Beck F, Jehan F, Domon-Dell C, et al. Multiple regulatory regions control the complex expression pattern of the mouse Cdx2 homeobox gene. Gastroenterology. 2008;135:1238–47,47 e1-3. 10.1053/j.gastro.2008.06.045 18655789

[103] Fong YW, Inouye C, Yamaguchi T, Cattoglio C, Grubisic I, Tjian R. A DNA repair complex functions as an Oct4/Sox2 coactivator in embryonic stem cells. Cell. 2011;147:120–131. 21962512 10.1016/j.cell.2011.08.038 PMC3216680

[104] Mutoh H, Hayakawa H, Sashikawa M, Sakamoto H, Sugano K. Direct repression of Sonic Hedgehog expression in the stomach by Cdx2 leads to intestinal transformation. Biochem J. 2010;427:423–434. 20199401 10.1042/BJ20091177

[105] Ramalho-Santos M, Melton DA, McMahon AP. Hedgehog signals regulate multiple aspects of gastrointestinal development. Development. 2000;127:2763–2772. 10821773 10.1242/dev.127.12.2763

[106] Engevik AC, Feng R, Yang L, Zavros Y. The acid-secreting parietal cell as an endocrine source of Sonic Hedgehog during gastric repair. Endocrinology. 2013;154:4627–4639. 24092639 10.1210/en.2013-1483 PMC3836061

[107] Schumacher MA, Feng R, Aihara E, Engevik AC, Montrose MH, Ottemann KM, Zavros Y. Helicobacter pylori-induced Sonic Hedgehog expression is regulated by NFkappaB pathway activation: the use of a novel in vitro model to study epithelial response to infection. Helicobacter. 2015;20:19–28. 25495001 10.1111/hel.12152 PMC4871133

[108] GR VDB, JC H, Nielsen C, Xu C, FJ TK, Glickman J,van Deventer SJ, Roberts DJ, Peppelenbosch MP. Sonic hedgehog expression correlates with fundic gland differentiation in the adult gastrointestinal tract. Gut. 2002;51:628–633. 12377798 10.1136/gut.51.5.628 PMC1773421

[109] Shiotani A, Iishi H, Uedo N, Ishiguro S, Tatsuta M, Nakae Y, Kumamoto M, Merchant JL. Evidence that loss of sonic hedgehog is an indicator of Helicobater pylori-induced atrophic gastritis progressing to gastric cancer. Am J Gastroenterol. 2005;100:581–587. 15743355 10.1111/j.1572-0241.2005.41001.x

